# Systemic but not topical TRAIL-expressing mesenchymal stem cells reduce tumour growth in malignant mesothelioma

**DOI:** 10.1136/thoraxjnl-2013-204110

**Published:** 2014-02-24

**Authors:** Elizabeth K Sage, Krishna K Kolluri, Katrina McNulty, Sofia Da Silva Lourenco, Tammy L Kalber, Katherine L Ordidge, Derek Davies, Y C Gary Lee, Adam Giangreco, Sam M Janes

**Affiliations:** 1 Division of Medicine, Lungs for Living Research Centre, University College London, London, UK; 2 Division of Medicine and Institute of Child Health, UCL Centre of Advanced Biomedical Imaging, University College London, London, UK; 3 Flow Cytometry Laboratory, Cancer Research UK, London Research Institute, London, UK; 4 School of Medicine and Pharmacology, University of Western Australia, Sir Charles Gairdner Hospital, Perth, Australia

**Keywords:** Mesothelioma, Asbestos Induced Lung Disease, Lung Cancer, Occupational Lung Disease

## Abstract

Malignant pleural mesothelioma is a rare but devastating cancer of the pleural lining with no effective treatment. The tumour is often diffusely spread throughout the chest cavity, making surgical resection difficult, while systemic chemotherapy offers limited benefit. Bone marrow-derived mesenchymal stem cells (MSCs) home to and incorporate into tumour stroma, making them good candidates to deliver anticancer therapies. Tumour necrosis factor-related apoptosis-inducing ligand (TRAIL) is a pro-apoptotic molecule that selectively induces apoptosis in cancer cells, leaving healthy cells unaffected. We hypothesised that human MSCs expressing TRAIL (MSCTRAIL) would home to an in vivo model of malignant pleural mesothelioma and reduce tumour growth. Human MSCs transduced with a lentiviral vector encoding TRAIL were shown in vitro to kill multiple malignant mesothelioma cell lines as predicted by sensitivity to recombinant TRAIL (rTRAIL). In vivo MSC homing was delineated using dual fluorescence and bioluminescent imaging, and we observed that higher levels of MSC engraftment occur after intravenous delivery compared with intrapleural delivery of MSCs. Finally, we show that intravenous delivery of MSCTRAIL results in a reduction in malignant pleural mesothelioma tumour growth in vivo via an increase in tumour cell apoptosis.

**Key messages**

**What is the key question?**

- Can TRAIL delivered by mesenchymal stem cells (MSCs) be an effective therapeutic option in malignant pleural mesothelioma?

**What is the bottom line?**

- When delivered systemically, MSCs expressing TRAIL successfully incorporate into malignant pleural mesothelioma and induce cancer cell death.

**Why read on?**

- This is the first paper that uses both bioluminescent and fluorescent in vivo imaging to show MSC homing and infiltration into malignant pleural mesothelioma and delivery of TRAIL to cause a reduction in tumour burden.

## Introduction

Malignant mesothelioma (MM) is a rare but devastating malignancy found most commonly within the pleura. It is largely caused by asbestos exposure^1^ and the mortality rate is increasing with >2300 deaths per year in the UK.^2^ Current treatment options are poor, and first-line chemotherapy with cisplatin and pemetrexed offers an average survival of 12 months.^3^ The role of radical surgery is controversial with the only large-scale clinical trial showing a trend to worse outcomes in patients undergoing extrapleural pneumonectomy.^4^ Because of the resistance of malignant pleural mesothelioma (MPM) to conventional treatments, new therapies are desperately needed. Most chemotherapy agents act by inducing tumour cell apoptosis via the intrinsic apoptotic pathway; however, MPM is known to be resistant to activation of this pathway, so interest has turned to activation of the extrinsic apoptotic pathway.^5^

Tumour necrosis factor (TNF)-related apoptosis-inducing ligand (TRAIL) is a type II transmembrane protein and member of the TNF superfamily. It binds via two active transmembrane death receptors, DR4 and DR5, triggering the caspase cascade resulting in apoptosis. TRAIL is an exciting anticancer molecule as it induces cell death in cancer cells without affecting healthy cells.^6^ Phase I clinical trials looking at the use of both recombinant TRAIL (rTRAIL)^7^ and monoclonal antibodies to the TRAIL death receptors, DR4 and DR5, have shown promising results.^8^ ^9^ However, there are problems with both of these treatment options. The half-life of rTRAIL is short at 32 min, meaning multiple infusions are needed to deliver therapeutic dose systemic therapy.^6^ While monoclonal antibodies have the advantage of a receptor-specific high-affinity binding enabling a prolonged half-life compared with recombinant TRAIL, this specificity may be problematic when looking for a therapeutic effect as there are two active TRAIL receptors and it is not known which receptor is more important for apoptotic signalling. This is a potential explanation for the disappointing results with these agents in clinical trials.^10^ ^11^

Bone marrow-derived mesenchymal stem cells (MSCs) are attractive candidates as vectors for anticancer therapies for multiple reasons. In vitro migration studies have demonstrated MSC migration towards both tumour cells and their conditioned media^12^ ^13^ while in vivo MSCs have been shown to incorporate into and persist in tumours following systemic administration in a wide variety of tumour models, including lung metastases^14^ and glioma.^15^ Multiple delivery routes are also effective for MSC therapy, including intravenous, direct intramyocardial^16^ and intraperitoneal delivery.^17^ While multiple growth factors and chemokines have been postulated to be important in MSC homing, the precise mechanism by which MSCs accumulate within tumours is poorly understood.^18^

In this study, we show for the first time that MSCs expressing TRAIL (MSCTRAIL) induce apoptosis in MPM cells in vitro and that MSCs home to and incorporate into tumours in vivo when delivered via both intrapleural and intravenous routes. Furthermore, we demonstrate that intravenous MSCTRAIL delivery causes a significant reduction in tumour growth in an in vivo model of MPM through a mechanism involving increased intratumoural MSC retention and tumour cell apoptosis.

## Materials and methods

### Cell culture

Tissue culture reagents were purchased from Invitrogen (Paisley, UK) unless otherwise stated. Human adult bone marrow-derived MSCs were purchased from Tulane University and cultured in α-MEM with 16% fetal bovine serum (FBS), 4 mM L-glutamine with 50 U/mL penicillin and 50 µg/mL streptomycin. MSCs transduced with a Tet-inducible plasmid had FBS replaced with Tet-system approved FBS (Clontech, Paris, France). Human MPM cell lines (MSTO-211H, H28, H2052, ONE58, JU77 and LO68) and the benign mesothelial cell line Met5A were a kind gift from Professor Bruce Robinson (University of Western Australia) and were cultured in Dulbecco's Modified Eagle Medium with 10% FBS, 4 mM L-glutamine with 50 U/mL penicillin and 50 µg/mL streptomycin.

### Lentiviral vectors

TRAIL-IRES-eGFP lentivirus vector was produced as previously described.^14^ Luciferase plasmid, pLIONII-HYG-Luc2YFP, was a gift from Dr Stephen Goldie (Cancer Research Institute, Cambridge), and lentivirus was produced using calcium phosphate transfection as described.^19^

MSCs were transduced with the TRAIL-IRES-eGFP lentivirus as previously described.^14^ Human TRAIL protein production with and without TRAIL activation in both cell supernatant and lysates was confirmed by ELISA (R&D Systems). MSTO-211H and H28 cells were transduced with pLIONII-HYG-Luc2YFP and selected with hygromycin 200 µg/mL until a pure population was achieved.

### Flow cytometry for TRAIL receptors

MPM cells were harvested and resuspended at 1×10^6^ cells/mL, then incubated with antibodies against the four TRAIL receptors or an isotype control, followed by biotinylated secondary goat antimouse IgG1 and streptavidin phycoerythrin (PE). Flow cytometry was performed to detect PE.

### In vitro co-culture experiments

MSCTRAIL cells were plated in a 1:1 ratio with human MPM cells, and apoptosis and cell death were determined as described in online supplementary materials and methods.

### In vivo pleural mesothelioma model

All animal studies were approved by the University College London Biological Services Ethical Review Committee and licensed under the UK Home Office regulations and the Evidence for the Operation of Animals (Scientific Procedures) Act 1986 (Home Office, London, UK). Eight-week-old female NOD/SCID mice were purchased from Harlan, kept in individually ventilated cages under specific pathogen-free conditions and had access to sterile-irradiated food and autoclaved water ad libitum.

To create pleural tumours, mice were anaesthetised using 2% isofluorane, the right thoracic wall was shaved and cleaned with alcohol. A 5 mm incision was made on the right chest wall, and a right anterolateral thoracotomy was performed in the fourth intercostal space, followed by installation of luciferase-transduced MSTO-211H (MSTO-211HLuc) cells in 100 µL phosphate-buffered saline (PBS). MSCs were either delivered intrapleurally as described or intravenously using the lateral tail vein. Animals were weighed twice weekly, and bioluminescent imaging of tumour burden was performed twice weekly. Tumours were allowed to develop until mice reached 20% weight loss or showed signs of distress.

### In vivo imaging of MSC homing

8×10^4^ MSTO-211HLuc were delivered intrapleurally and tumours were left to develop for 5 days. 1×10^6^ untransduced MSCs were labelled using the fluorescent lipophilic dyes, DiI (1,1′-dioctadecyl-3,3,3′3′-tetramethylindocarbocyanine perchlorate) and DiR (3,3,3′,3′ tetramethylindotricarbocyanine iodide) according to manufacturer's instructions and delivered either by intravenous or intrapleural injections 5 days post-tumour cell injection. Bioluminescence and fluorescence were determined using an in vivo imaging system (IVIS Lumina, Caliper Life Sciences) as described in online supplementary methods. At study termination, mice were killed and tumour samples were digested for flow cytometry and fixed for histochemical analysis.

### Flow cytometry analysis of tumours

Tumours were identified using open cavity bioluminescent imaging, removed and digested in a solution containing RPMI-1640 (Invitrogen) with 1 mg/mL collagenase (Sigma) and DNase I (Roche; 10 µg/mL) for 1 h at 37°C. Red blood cells were lysed with red blood cell lysis buffer (Sigma) for 1 min, neutralised with RPMI and filtered. Flow cytometry (LSR Fortessa, Beckton Dickinson) was performed for YFP to detect tumour cells and DiR to detect MSCs.

### In vivo therapeutic effect of MSCTRAIL

Pleural tumours were established as previously described. Tumours were left to develop for 5 days and for intravenous delivery 1×10^6^ MSCTRAIL or untransduced MSCs suspended in PBS or PBS alone were delivered intravenously on days 5, 9, 12, 15 and 18. For intrapleural delivery, the same protocol was followed except that animals received intrapleurally delivered MSCTRAIL, untransduced MSCs or PBS alone on days 5, 9, 12, 15 and 18. All animals received doxycycline at 2 mg/mL in sterile water containing 3% sucrose. Tumour growth was monitored via bioluminescent imaging twice weekly using IVIS and tumour burden was determined as described above. Animals were injected with 10 mg/kg 5-bromo-2′-deoxyuridine (BrdU; Invitrogen) 1 h prior to sacrifice. All tumour tissue and lungs were removed and weighed prior to fixation and fixed overnight in 10% neutral buffered formalin (Roche) for histology.

### Immunohistochemistry

Samples were processed as described in online supplementary methods. Calretinin antibody (Abcam), WT1 antibody (Upstate Cell Signaling Solutions, New York, USA), TRAIL, DR5 antibodies (ProSci) and Luciferase antibodies (rabbit polyclonal; Abcam) were used as primary antibodies. BrdU-positive and TUNEL-positive cells were quantified using Volocity software.

### Statistics

Statistical analysis was performed using GraphPad Prism V.4 (GraphPad Software). In vivo experiments with multiple groups were analysed using repeated measures ANOVA, and single-group data were assessed using Student t test. All in vitro experiments were performed in triplicate unless specified.

## Results

### Characterisation of cells and transduction of MSCs

**Figure 1:**
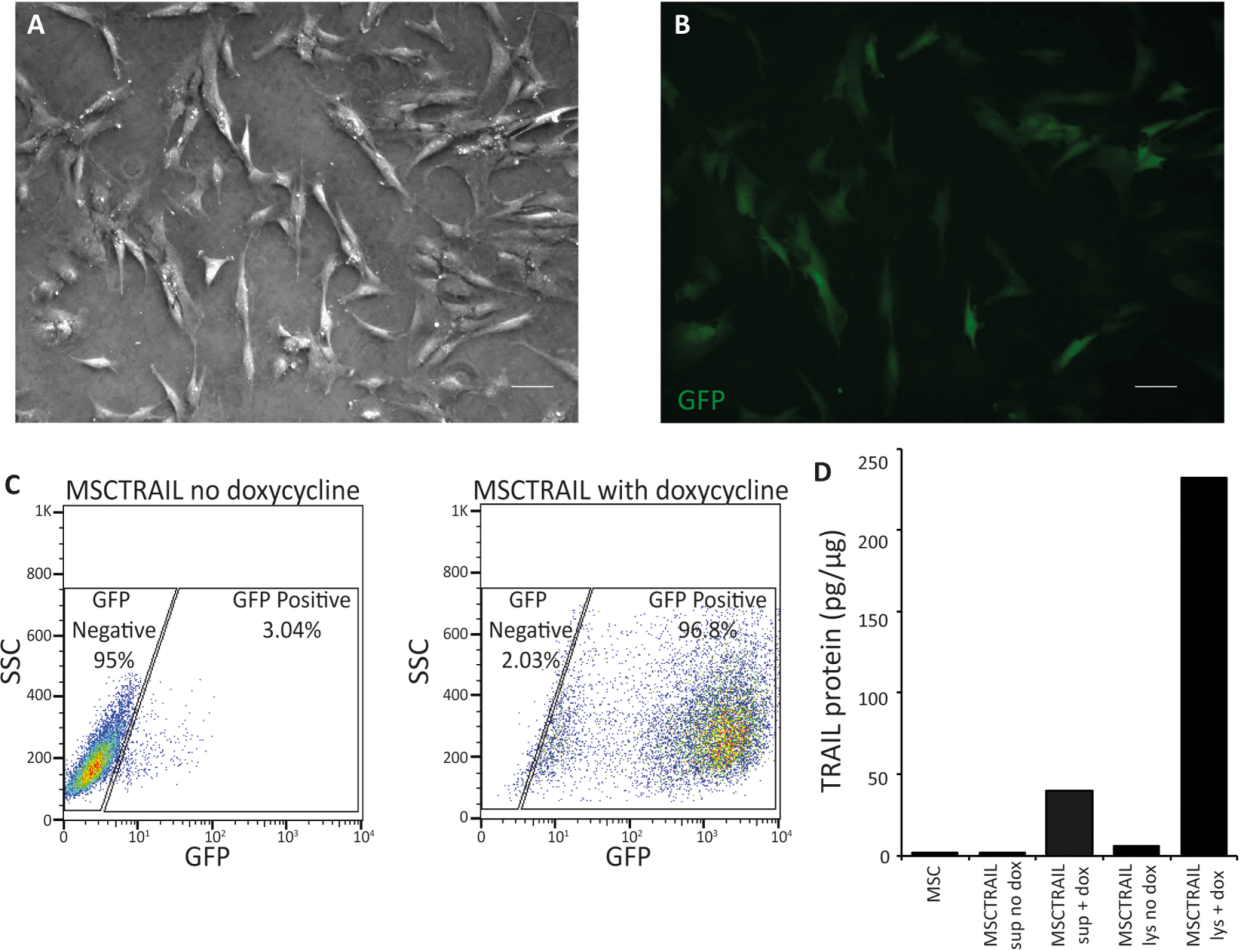
MSC transduction. (A) Bright field and (B) fluorescence microscopy to confirm GFP expression following transduction of MSC with TRAIL-IRES-eGFP lentivirus and activation with doxycycline (10 µg/mL) (magnification, ×5; bar 20 µm). (C) Flow cytometry plots confirming efficiency of MSC transduction following TRAIL activation with doxycycline and (D) a TRAIL ELISA of MSC cell supernatant and lysate demonstrating the production of TRAIL protein in cell lysates following MSCTRAIL activation with doxycycline. There is minimal TRAIL production following MSC transduction in the absence of doxycycline and low levels in cell supernatant from activated MSCTRAIL. MSC, mesenchymal stem cell; MSCTRAIL, MSCs expressing TRAIL; TRAIL, tumour necrosis factor-related apoptosis-inducing ligand.

The ability of MSCs to differentiate into fat and bone was confirmed along with their colony-forming efficiency as previously described.^14^ MSC transduction with TRAIL-IRES-eGFP under the control of a tetracycline-dependent promoter was successful (figure 1A,B), and transduction efficiency was >96% following activation with doxycycline (figure 1C). ELISA confirmed high TRAIL expression in MSC cell lysates following TRAIL activation with doxycycline but low levels in cell lysates from inactivated MSCs and cell supernatants (figure 1D).

Immunocytochemistry confirmed both calretinin and Wilms Tumour antigen 1 (WT1) in all MPM cell lines (see online supplementary figure S1A–F), and they all possessed death receptor 5 (DR5) (see online supplementary figure S1G), the receptor responsible for the majority of TRAIL signalling. There was no correlation between the mean fluorescence intensity of DR5 staining and the sensitivity of MPM to MSCTRAIL (see online supplementary figure S1H). Met5A is shown as a normal mesothelial control.

### In vitro co-culture experiments demonstrate variable sensitivity of MPM cells to MSCTRAIL

**Figure 2:**
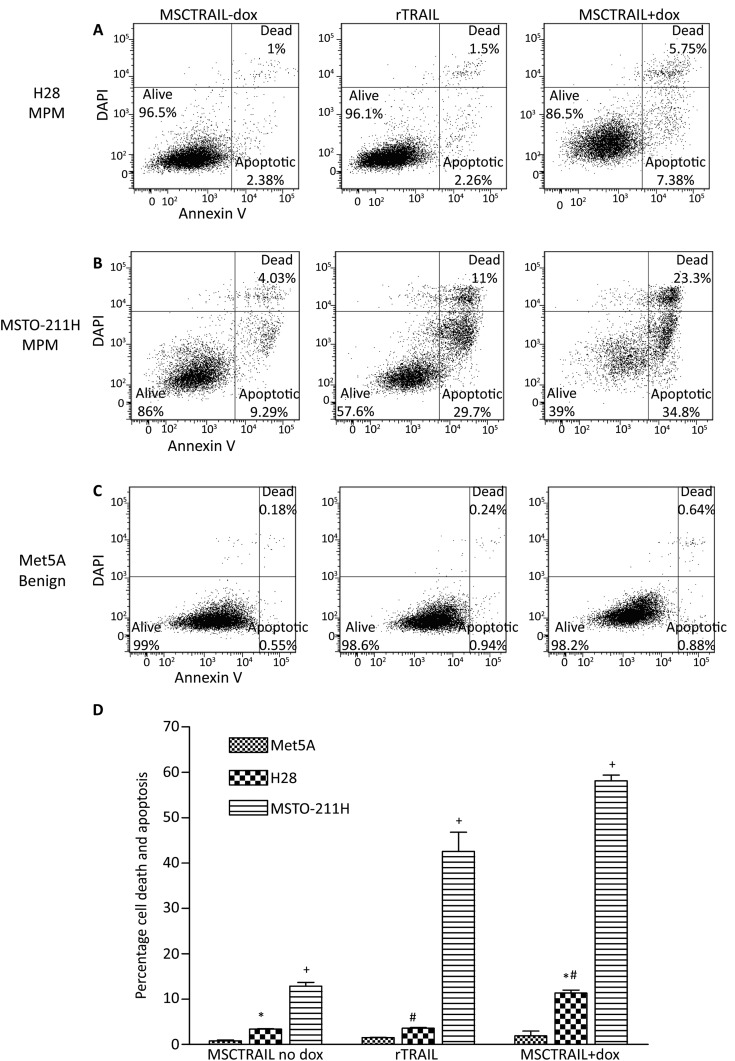
Human MPM exhibit variable in vitro sensitivity to rTRAIL and MSCTRAIL. Flow cytometry plots showing increased apoptosis and cell death in (A) H28 MPM when treated with rTRAIL and MSCTRAIL+dox and (B) in MSTO-211H MPM cell line with MSCTRAIL+dox compared with rTRAIL or MSCTRAIL no dox. Control benign mesothelial cells Met5A, (C) show no significant apoptosis or cell death when treated with rTRAIL or MSCTRAIL+dox. Apoptosis and death were determined using annexin V and DAPI staining, respectively. (D) Flow cytometry data from co-culture experiments showing an increase in apoptosis and death in both H28 and MSTO-211H following treatment with rTRAIL and MSCTRAIL. (+p<0.0001; *p=0.001; #p=0.007). MPM, malignant pleural mesothelioma; MSCTRAIL, mesenchymal stem cells expressing TRAIL; TRAIL, tumour necrosis factor-related apoptosis-inducing ligand.

MPM tumour cell lines were co-cultured for 48 h with rTRAIL or MSCTRAIL with doxycycline to activate TRAIL production. Five out of six cell lines (83%) showed sensitivity to TRAIL. H28 cells showed a significant increase in apoptosis and death when treated with MSCTRAIL compared with either rTRAIL (3.6±0.1% vs 11.3±0.7%, p=0.007) or inactivated MSCTRAIL (3.4±0.1% vs 11.3±0.7%, p=0.001) (figure 2A,D). MSTO-211H cells were sensitive to treatment with rTRAIL showing over 40% cell death compared with inactivated MSCTRAIL (42.6±4.2% vs 12.9±0.8%, p<0.0001), which increased to over 58% when treated with MSCTRAIL (12.9±0.8% vs 58.2±1.2%, p<0.0001) (figure 2B,D). In Met5A, there was no significant increase in apoptosis and death when treated with either rTRAIL or MSCTRAIL compared with inactivated MSCTRAIL (1.5±0.2% and 1.9±1.1%, respectively, vs 0.8±0.05%, p=0.283 and p=0.285, respectively) (figure 2C,D). ONE58, JU77, H2052 and LO68 were also tested and were sensitive to MSCTRAIL (see online supplementary Fig S2A).

To confirm that cell death was not caused by treatment with doxycycline, MPM cells were incubated for 48 h with 10 µg/mL doxycycline, harvested and stained for annexin V and DAPI as before. Flow cytometry showed no significant increase in cell death or apoptosis in any of the MPM cell lines tested (see online supplementary figure S2B).

### Bioluminescence is suitable for monitoring longitudinal MPM tumour growth and a quantitative end point of tumour burden

**Figure 3:**
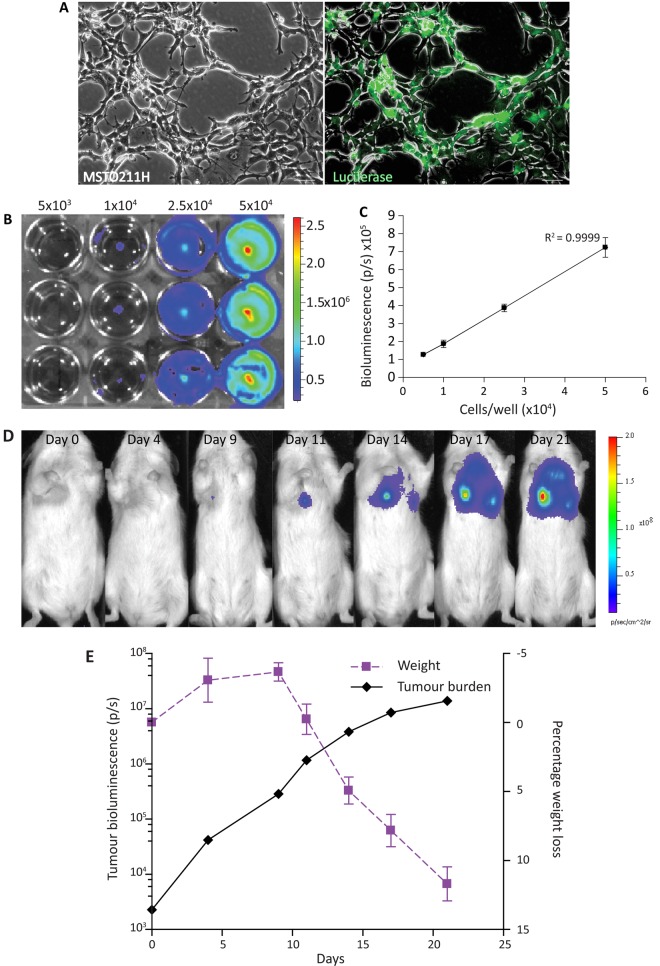
Luciferase-transduced MSTO-211H cells injected intrapleurally can be tracked longitudinally and correspond to tumour growth. (A) Human MPM cells MSTO-211H were transduced with pLIONII-HYG-Luc2YFP and selected with hygromycin (200 µg/mL) until a pure population was achieved (phase contrast, left panel and immunofluorescence of luciferase transduced, right panel) (magnification 5×; bar 60 µm). (B) MSTO211HLuc were plated in increasing numbers in a 12-well plate and imaging was performed once cells were adherent. (C) Graph of bioluminescent signal with increasing cell number showing good correlation between increasing signal and increasing cell number (represented as average±SEM). (D) IVIS images demonstrate that intrapleural delivery of MSTO-211HLuc cells results in an increase bioluminescent signal over time. (E) The most rapid increase in tumour growth occurs within the first 10 days following tumour cell inoculation. Weight loss does not occur until tumour growth slows, suggesting it is a late marker of disease. MPM, malignant pleural mesothelioma.

MSTO-211H cells were transduced with a luciferase-YFP (MSTO-211HLuc) lentiviral vector and selected using hygromycin (200 µg/mL) to ensure a pure population (figure 3A) and increasing cancer cell numbers correlated well with increasing bioluminescent signal (figure 3B,C).

To determine the kinetics of tumour growth in vivo, 8×10^4^ MSTO-211HLuc cells were injected intrapleurally and tumour growth was monitored. Bioluminescence images showed increasing signal over 21 days (figure 3D,E). MSTO-211H cells were used as they are known to be tumourigenic in vivo while H28 are not.^20^

### Intrapleurally and intravenously delivered MSCs home to MPM in vivo

**Figure 4:**
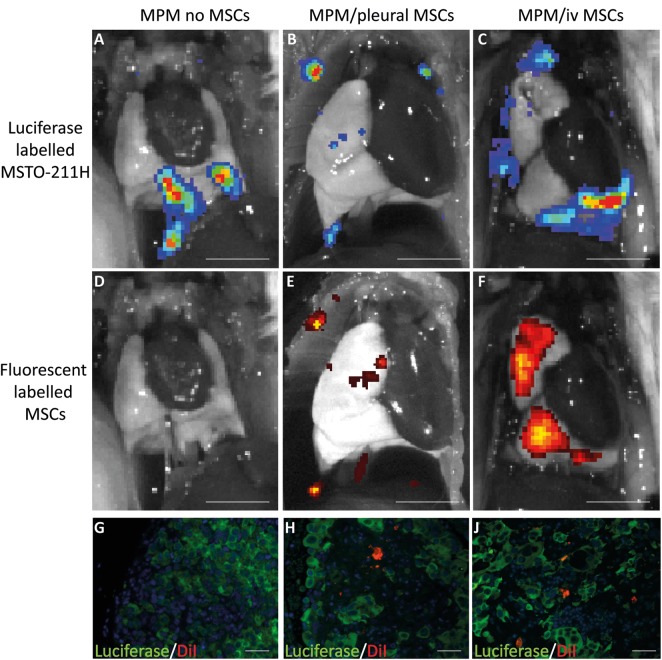
Human MSCs home to an in vivo model of MPM when delivered both intrapleurally and intravenously. (A–C) IVIS images of animals with intrapleural bioluminescent MSTO-211HLuc tumour cells. (D) Lack of fluorescent signal in control animal with no DiR-labelled MSCs while animals receiving (E) pleural MSCs and (F) intravenous MSCs show co-localisation of fluorescent MSCs at the sites of tumour (scale bar 5 mm). (G) Immunofluorescence images confirm the absence of DiI-labelled MSCs (red) within luciferase positive tumour, while MSCs are visible within the luciferase-positive tumours following both (H) intrapleural and (J) intravenous MSC delivery (magnification ×20; bar 60 µm). MPM, malignant pleural mesothelioma; MSC, mesenchymal stem cell.

Tumours were established as described in the methods with 1×10^6^ DiI-labelled MSCs injected into mice either intrapleurally or intravenously. Animals were imaged immediately after injection and 48 h later to determine the location of both MSCs and tumours. Tumours were clearly visible on bioluminescent imaging (figure 4A–C), and fluorescence demonstrated MSC localisation at the site of the tumours (figure 4E,F). No animals developed pleural effusions so signal is representative of solid tumours. Control animals receiving no MSCs showed no fluorescent signal, confirming that any signal detected was representative of MSCs and not luciferase-YFP (figure 4D). Immunofluorescence confirmed that DiI-labelled MSCs were located within the tumour stroma when delivered both intrapleurally and intravenously (figure 4G–J). No fluorescent signal was seen outside the lungs, suggesting that MSCs did not reach other organs and there was no evidence of toxicity.

### MSCTRAIL causes a reduction in tumour growth when delivered intravenously but not intrapleurally

**Figure 5:**
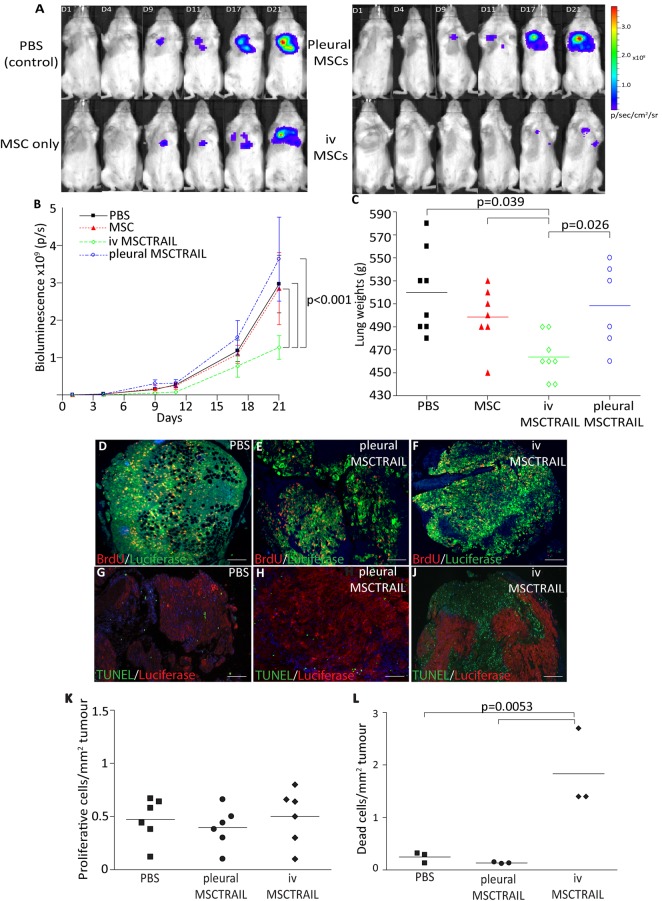
MSCTRAIL reduce the growth of MPM when delivered intravenously. (A) IVIS images of representative animals from each experimental group showing reduced bioluminescent signal in animals treated with intravenous MSCTRAIL. (B) Line graph to demonstrate a reduction in total photon count (p/s) seen in animals in the intravenous MSCTRAIL-treated group over 21 days compared with MSC delivery alone, PBS or intrapleural MSCTRAIL. Photon count was determined using dedicated regions of interest around the whole body to include both pleural tumour burden and any distant metastatic deposits (Living Image Software, Caliper LifeSciences; p<0.001). (C) Dot plot to show a reduction in lung weights with intravenous MSCTRAIL treatment compared with all other treatment groups (p<0.05). Immunofluorescence showing proliferating cells (red) and luciferase-positive tumour cells (green) in tumours treated with (D) PBS, (E) intrapleural-delivered MSCTRAIL and (F) intravenous-delivered MSCTRAIL. (G) Representative immunofluorescence showing TUNEL-positive apoptotic cells (green) within pleural tumours (red) in animals treated with (G) PBS, (H) pleural MSCTRAIL and (J) increased apoptosis in tumours treated with intravenous MSCTRAIL (magnification 4×, bar 60 µm). (K) Proliferating cells per tumour area were quantified (Volocity Software) with no significant difference in the number of BrdU-positive cells between the treatment groups. (L) TUNEL-positive cells per tumour area were quantified (Volocity Software), and MSCTRAIL-treated animals showed increased levels of apoptosis within tumours compared with all other groups (p=0.0053). MPM, malignant pleural mesothelioma; MSC, mesenchymal stem cell; MSCTRAIL, MSCs expressing TRAIL; PBS, phosphate-buffered saline; TRAIL, tumour necrosis factor-related apoptosis-inducing ligand.

Tumours were established as described and 1×10^6^ MSCTRAIL or untransduced MSC or 100 µL PBS were delivered intravenously on days 5, 9, 12, 15 and 18. IVIS imaging demonstrated a significant reduction in tumour growth in the MSCTRAIL group compared with the PBS and untransduced MSC group (p<0.001, repeated measures ANOVA; figure 5A,B). There was also a significant reduction in lung weights in MSCTRAIL-treated mice (p<0.0391; figure 5C).

To determine whether a similar effect was seen with topically delivered MSCTRAIL, the experiment was repeated using intrapleural delivery of 1×10^6^ MSCTRAIL or untransduced MSC or PBS. In this experiment, there was no significant reduction in tumour growth or lung weights compared with the PBS and untransduced MSC-treated groups.

Histopathological analysis showed no significant difference in tumour cell proliferation (figure 5D–F and K), but there was a significant increase in apoptosis in tumours from the intravenous MSCTRAIL-treated group (p<0.0053; figures 5G–J and L), suggesting that intravenous MSCTRAIL cells reduce tumour size by inducing apoptosis. Representative H&E sections from tumours treated with PBS, intrapleural MSCTRAIL and intravenous MSCTRAIL (see online supplementary figure S3) show the presence of pyknotic nuclei and dead cells in the intravenous MSCTRAIL-treated tumour.

### Intravenous-delivered MSCs incorporate into tumours in greater numbers than intrapleural- delivered MSCs

**Figure 6:**
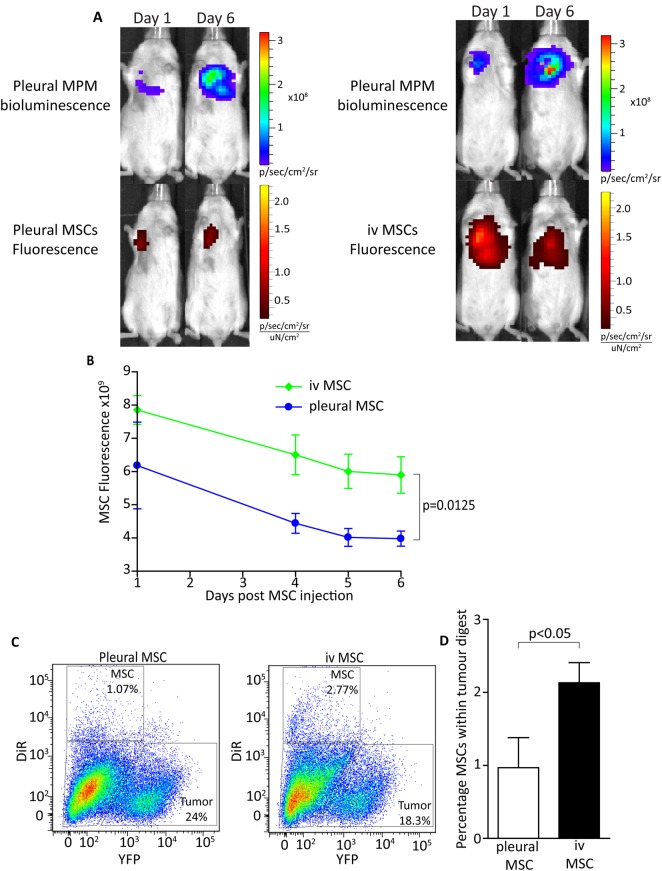
Intravenous-delivered MSCs are incorporated into tumours in greater numbers than when delivered intrapleurally. (A) IVIS images to show established bioluminescent MPM tumours and corresponding fluorescence from DiR-labelled MSCs on days 1 and 6 following MSC injection. Intravenous-delivered MSCs show a higher fluorescent signal on day 1 and day 6 following injection compared with intrapleural-delivered MSCs. (B) Fluorescent signal was quantified and MSCs delivered intravenously showed a higher signal on day 1 compared with cells delivered intrapleurally, which persisted until day 6 (p=0.0125). (C) Tumours were removed and digested for flow cytometry, which revealed a higher percentage of DiR-stained MSCs in tumours given intravenous MSCs than in those given intrapleural MSCs. (D) Bar chart to show a significant increase in DiR-stained MSCs in tumour digests following intravempis delivery compared with pleural delivery (p<0.0412). MPM, malignant pleural mesothelioma; MSC, mesenchymal stem cell.

To determine why MSCTRAIL is only effective when delivered intravenously, mice were given 2×10^5^ MSTO-211HLuc intrapleurally and tumours were left to establish for 10 days. 1×10^6^ DiR-stained MSCs stained were injected either intravenously or intrapleurally and imaging was performed daily. Tumours were successfully established and fluorescent MSCs were clearly visible following both routes of delivery (figure 6A). There was a significant difference in signal 24 h post-MSC injection (p=0.0125; figure 6B), which was maintained throughout the imaging period, suggesting that MSCs incorporate into tumours in greater numbers when delivered intravenously compared with intrapleurally. Flow cytometry of tumour digests confirmed a greater percentage of MSCs in the tumours receiving intravenous MSCs (figure 6C,D), although the overall percentage was low compared with the number of tumour stromal cells and lung cells.

## Discussion

In this study, we have shown that MSCs engineered to express TRAIL can induce death in multiple MPM cell lines in vitro and are more efficient at killing than recombinant TRAIL. We have also demonstrated that MSCs migrate to MPM tumours in vivo when delivered both intravenously and intrapleurally. However, only intravenous-delivered MSCTRAIL causes a significant reduction in tumour growth and the difference in efficacy is likely due to a higher number of MSCs engrafting within the tumours causing increased apoptosis.

Using MSCs as vectors for gene therapy is becoming increasingly common as they are easy to extract from bone marrow, are highly expandable and readily transducible with viral vectors.^21^ Once modified they maintain their stem cell properties^22^ and can be injected into a recipient without provoking an immune response.^23^ Clinical trials using allogeneic and autologous MSCs in cardiovascular and respiratory diseases have shown no adverse events or immunological reactions,^24^ ^25^ but as yet there are no clinical trials using engineered MSCs in cancer. The issue of immunological reactions will clearly need to be monitored for in any phase I clinical trial with engineered MSCs. MSCs home to multiple tumour types in vitro and in vivo, and while the precise mechanism of this has not been clearly established multiple cytokine/receptor pairs have been investigated.^26^ In our study, we show that MSCs home to MPM when delivered both intravenously and intrapleurally and are incorporated within the tumour tissue. These properties enable delivery of high-dose-targeted cancer therapy directly to the site of the tumour while reducing off-target effects, making it a clinically attractive option.

There is some concern that exogenously delivered MSCs have an unpredictable effect on tumour biology with different in vivo models suggesting pro-tumourigenic or antitumourigenic properties,^27^ but MSCs genetically modified to express pro-apoptotic molecules ensure an antitumourigenic effect.^28^ This is in line with our results where MSCs alone had no pro-tumourigenic effect and intravenous MSCTRAIL had an antitumourigenic effect. TRAIL is an exciting prospect for cancer therapy because of its ability to selectively target cancer cells without killing healthy cells; however, its short half-life^6^ means repeated high-dose systemic treatment would be required to produce a significant local effect. While human MPM cell lines express both DR4 and DR5 receptors with higher levels of DR5 than DR4, this expression does not correlate with TRAIL sensitivity.^29^ There are currently no biomarkers to predict sensitivity to TRAIL. The efficacy of rTRAIL-mediated killing of MPM has been assessed in vitro in combination with multiple other therapies,^30^ ^31^ but not as a single agent or in vivo models. MSCTRAIL is known to eliminate or reduce tumour growth in other in vivo cancer models,^14^ ^15^ ^32^ but has not been assessed in MPM. It can kill the cancer stem cell-like populations of cancer cells that are thought to be resistant to current chemotherapies and are a postulated mechanism for tumour recurrence following treatment^33^ . Our results demonstrate for the first time that MPM cells are sensitive to treatment with MSCTRAIL and there is a greater level of cell death compared with treatment with rTRAIL in vitro. This treatment remains effective in vivo with a significant reduction in tumour burden when MSCTRAIL is delivered intravenously. During our experiments, the number of MSCTRAIL cells injected appears high relative to tumour cell inoculation. However, when the population doubling time of the MSTO-211H cells of 20 h is considered, the ratio of tumour cells to MSCTRAIL cells would be significantly lower.^34^

When using MSCs as therapeutic delivery vectors, treatment efficacy is related to the level of MSC accumulation at tumour sites. Our study shows that while MSC homing occurs regardless of route of delivery, the level of MSC accumulation following intravenous delivery is significantly higher than when delivered intrapleurally. The increase in apoptosis seen in the intravenous delivery model could be a direct effect of the tumour receiving a greater number of cells and hence a higher dose of TRAIL. The first step in MSC accumulation within tumours is adhesion to vascular endothelial cells, and multiple factors are involved in this process.^35^ ^36^ It may be that cells delivered intravenously have higher accumulation as they are delivered directly to endothelial cells, making adhesion more likely. Alternatively intrapleurally delivered cells may have lower intratumoural accumulation as their delivery is likely to be to areas that are poorly vascularised and hypoxic and MSCs are more likely to die when located within a hypoxic microenvironment.^37^

In conclusion, this study shows intravenous MSCTRAIL delivery causes a reduction in tumour growth in an in vivo MPM model. MSCs home to and incorporate into tumours using both intravenous and intrapleural delivery routes but greater numbers engraft when delivered intravenously. The therapeutic effect seen with intravenous delivery could be related to greater engraftment of MSCs within the tumour and is an important finding when considering the future therapeutic role of MSCTRAIL therapy in the clinic.

## Supplementary Material

Web supplement

Web figures
