# Supplementary material for: Systemic but not topical TRAIL-expressing mesenchymal stem cells reduce tumour growth in malignant mesothelioma
Source: Thorax. 2014 Feb 24;69(7):638–47. doi: 10.1136/thoraxjnl-2013-204110 (PMC4078753; doi:10.1136/thoraxjnl-2013-204110)
Supplement: Web supplement [file thoraxjnl-2013-204110-s1.pdf]

Systemic but not topical TRAIL-expressing Mesenchymal Stem Cells reduce tumour growth in Malignant Mesothelioma.

Elizabeth K Sage, Krishna K Kolluri, Katrina McNulty, Sofia Da Silva Lourenco, Tammy L Kalber, Katherine L Ordidge, Derek Davies, Y C Gary Lee, Adam Giangreco and Sam M Janes

Supplementary Materials and Methods

***In vitro* co-culture experiments.** MSCTRAIL cells were plated in a six-well plate in a 1:1 ratio with human malignant pleural mesothelioma cells (MPM) and allowed to adhere for 24 hours. Prior to plating MPM were stained with the lipophilic dialkylcarbicyanine membrane dye, DiI, according to manufacturer's instructions (Invitrogen), to allow this population to be identified by flow cytometry. Once cells were adherent media was changed for either standard media or in the case of TRAIL transgene activation with media containing doxycycline (10 µg/ml) and left for 48 hours.

Apoptosis and cell death were determined by flow cytometry (LSRII or LSR Fortessa, Becton Dickinson). All floating and adherent cells were harvested and stained with Annexin V-AF647 antibody (Invitrogen) and 4', 6-diamidino-2-phenylindole (DAPI; 200 µg/ml; Sigma). Experiments were performed in triplicate.

***In vivo* imaging of MSC homing.** $8x10^4$ luciferase transduced MSTO-211H (MSTO-211HLuc) were delivered into the right pleural cavity of NOD/SCID mice as described above. Animals were left for 5 days for tumours to develop and growth was monitored using optical bioluminescence determined by an *in vivo* imaging system (IVIS Lumina, Caliper Life Sciences). To detect bioluminescence D-luciferin (Regis Technologies) was administered via intraperitoneal injection (100 mg/kg body weight) 15 minutes prior to imaging. $1x10^6$ MSCs

were labelled using DiI and DiR according to manufacturer's instructions and delivered either by iv or ip injections 5 days post tumour cell injection. Fluorescence was determined by IVIS using a 745nm excitation wavelength and detected with an ICG filter. Both bioluminescent and fluorescent signal intensity was determined using dedicated regions of interest and quantified using Living Image software (Xenogen). At specified time points after MSC injection animals were imaged and sacrificed by an overdose of sodium pentobarbital. Lungs were exposed and imaged as above to determine the precise location of tumours and MSCs. Tumour samples were digested for flow cytometry analysis and fixed for histochemical analysis.

Immunohistochemistry. Fixed specimens were washed in 70% ethanol before being embedded in paraffin and cut into 3 µm sections for H&E staining. Calretinin antibody (Abcam), WT1 antibody (Upstate Cell Signaling Solutions, NY), TRAIL, DR5 antibodies (ProSci) and Luciferase antibodies (rabbit polyclonal; Abcam) were used as primary antibodies and detected with biotinylated secondary's and DAPI for nuclear localisation. TUNEL staining was performed according to manufacturer's instructions (Promega). Microscopy was performed using light (Olympus BX40) and fluorescent microscopes (Carl Zeiss, Axioskop 2 and Axioscope Lumar V12 Stereo). BrdU and TUNEL positive cells were quantified using Volocity software.
