# Supplementary material for: Systemic but not topical TRAIL-expressing mesenchymal stem cells reduce tumour growth in malignant mesothelioma
Source: Thorax. 2014 Feb 24;69(7):638–47. doi: 10.1136/thoraxjnl-2013-204110 (PMC4078753; doi:10.1136/thoraxjnl-2013-204110)

Supplementary Figures

Supplementary Figure 1. Human MPM Characterisation. A-C, immunofluorescence confirms the presence of calretinin and D-F WT1 antigen in human MPM cell lines H28 and MSTO211H and human benign mesothelial cell line Met5A (magnification x20, bar 40µm). G, Flow cytometry histograms of H28, JU77, MSTO-211H and ONE58 MPM cell lines showing the presence of the active TRAIL death receptor DR5 with relative absence of other death and decoy receptors. H, Scatter graph of percentage apoptosis against mean fluorescence intensity of the active TRAIL receptor DR5 showing no significant correlation between the levels of DR5 expression and sensitivity to TRAIL induced apoptosis (R^2=0.13).

Supplementary Figure 1

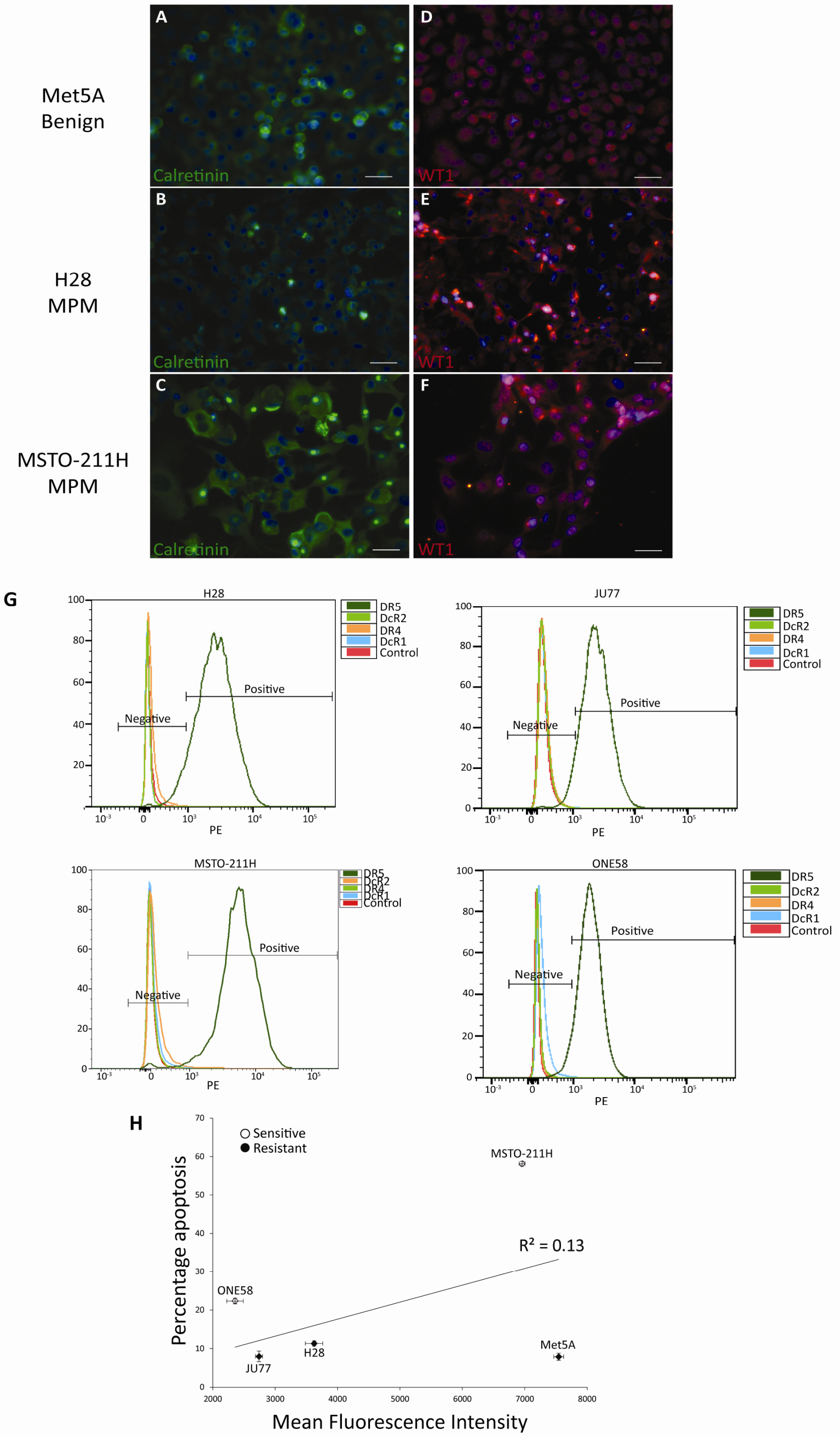

Supplementary Figure 2. Human MPM show variable sensitivity *in vitro* to rTRAIL and MSCTRAIL. A, ONE58 human MPM cells are very sensitive to both rTRAIL and MSCTRAIL when compared to MSCs alone and this is the only cell line where treatment with rTRAIL results in significantly higher apoptosis and death when compared to MSCTRAIL treatment. JU77 shows a significant increase in death and apoptosis when treated with rTRAIL compared to MSC. There is a further increase when comparing rTRAIL with MSCTRAIL treatment. H2052 shows a significant increase in cell death and apoptosis when treated with both rTRAIL and MSCTRAIL compared to MSC alone but no difference between the two TRAIL treatments. Finally LO68 is not sensitive to rTRAIL but does show significant increases in death and apoptosis when treated with MSCTRAIL (*, $p<0.05$; **, $p<0.001$; ***$p<0.0001$). B, MPM and benign mesothelial cells show no significant differences in death and apoptosis when treated with doxycycline 10 µg/ml when compared to those not exposed to doxycycline.

Supplementary Figure 2

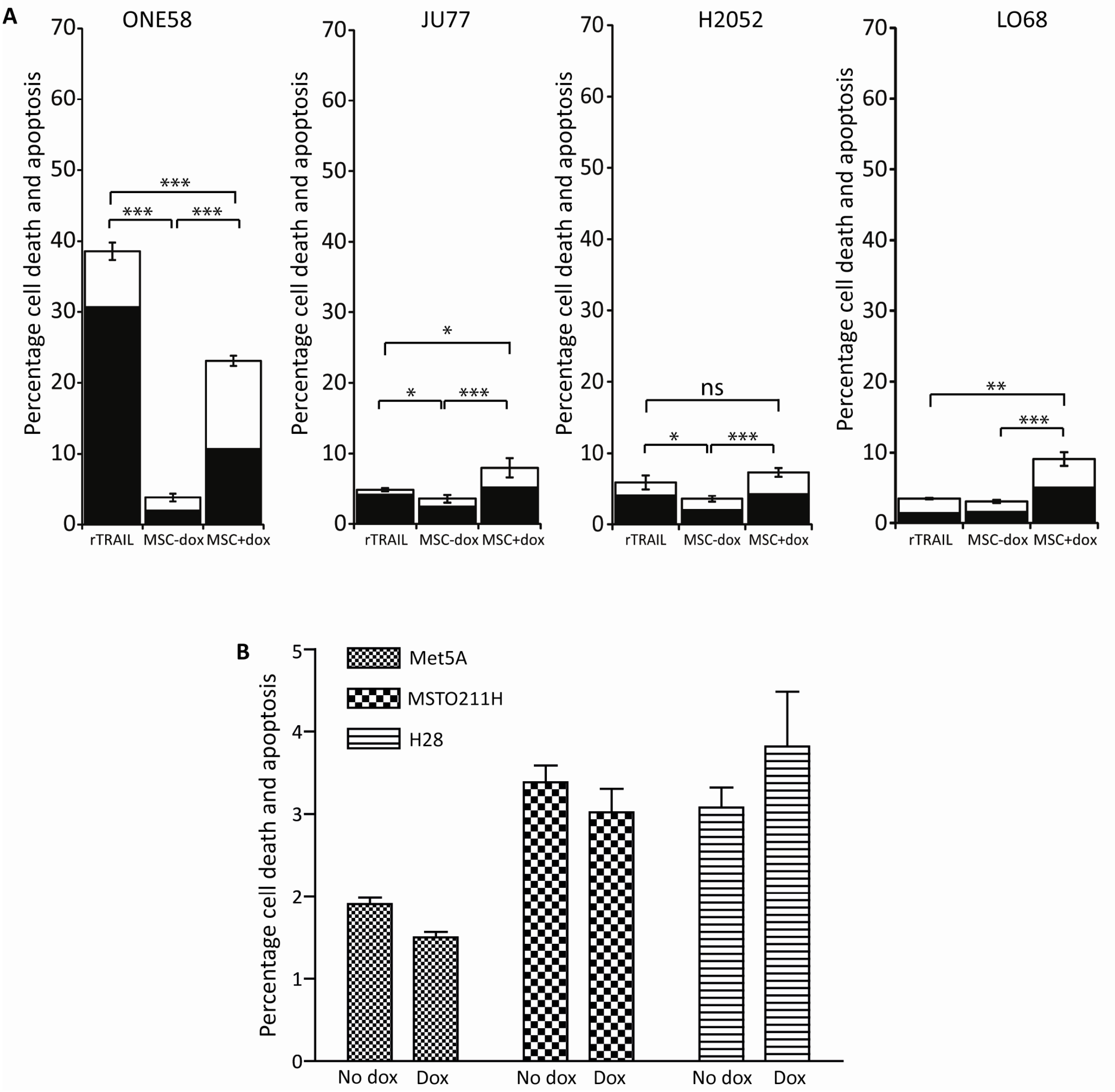

Supplementary Figure 3. H&E staining reveals pyknotic cells in tumours undergoing apoptosis. A, Representative H&E section of MPM tumour treated with PBS, B, pleural MSCTRAIL and C, iv MSCTRAIL (magnification 20x, scale bar 100 μm). Tumour treated with iv MSCTRAIL shows the presence of pyknotic nuclei consistent with cells undergoing apoptosis and areas of cell death.

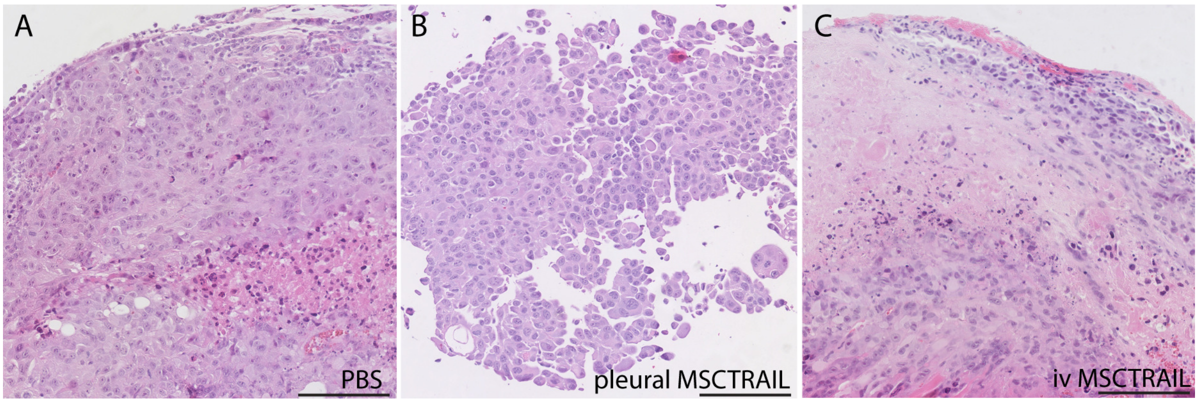

Supplement: Web figures [file thoraxjnl-2013-204110-s2.pdf]
